# miRNome analysis reveals mir-155-5p as a protective factor to dengue infection in a resistant Thai cohort

**DOI:** 10.1007/s00430-025-00821-7

**Published:** 2025-02-20

**Authors:** Isabelle Casadémont, Rubén Ayala-Suárez, Naphak Modhiran, Ahmed Tawfik, Matthieu Prot, Richard Paul, Etienne Simon-Lorière, Francisco Díez-Fuertes, Sukathida Ubol, José Alcamí, Anavaj Sakuntabhai

**Affiliations:** 1 Institut Pasteur, Université Paris-Cité, CNRS UMR 2000, 28 rue du Dr Roux, Paris, France; 2 AIDS Immunopathology Unit, Instituto de Salud Carlos III, Majadahonda, Spain; 3 Spanish Consortium for Research in Infectious Diseases (CIBERINFEC), Madrid, Spain; 4 Departamento de Biomedicina y Biotecnología, Universidad de Alcalá, Alcalá de Henares, Spain; 5 Faculty of Science, Department of Microbiology, Mahidol University, Bangkok, Thailand; 6 School of Chemistry and Molecular Biosciences, Faculty of Science, University of Queensland, Brisbane, Australia; 7 AIDS and HIV Infection Group (VIH-Clínic), Fundació de Recerca Clínic Barcelona, Institut d’Investigacions Biomèdiques August Pi i Sunyer (FRCB-IDIBAPS), Barcelona, Spain

**Keywords:** microRNA, miRNome, Dengue, RNA-Seq, Resistance, miR-155-5p

## Abstract

Dengue virus (DENV) is a global health threat, with approximately 390 million infections annually, ranging from mild dengue fever to severe dengue hemorrhagic fever and shock syndrome. MicroRNA (miRNA) are crucial post-transcriptional regulators which may regulate host resistance to DENV infection. This study aimed to identify miRNAs involved in natural resistance to DENV infection. Individuals from a dengue-endemic area were classified as susceptible (SD) or resistant (RD) according to their anti-DENV antibody status. RD individuals were seronegative despite high local DENV infection prevalence. Monocytes susceptibility to DENV infection was assessed in vitro. The miRNome profiles of the monocytes from 7 individuals per group were assessed upon mock or DENV-2 infection. The antiviral effect of differentially expressed miRNAs was analyzed using miRNA mimics in HeLa cells followed by infection with DENV-1, DENV-2, DENV-3, and DENV-4 serotypes. We performed RNA-seq on miRNA mimic-transfected cells to identify miRNA-targeted genes interacting with DENV proteins. Monocytes from RD individuals exhibit lower DENV-2 production in vitro. The miRNAs miR-155, miR-132-3p, miR-576-5p were overexpressed in monocytes from RD group upon DENV-2 infection. The transfection of miR-155-5p mimic reduced DENV infection and viral production in HeLa cells, regulating 18 genes interacting with DENV proteins and downregulating target genes involved in interferon response, TP53 regulation, apoptosis, and vesicle trafficking (e.g. *HSD17B12, ANXA2*). Therefore, we show that monocytes from RD individuals show a distinct miRNA expression profile and reduced viral production. In vitro miR-155-5p upregulation induces an antiviral state, revealing potential therapeutic targets to treat dengue.

**Supplementary Information:**

The online version contains supplementary material available at 10.1007/s00430-025-00821-7.

## Background

Dengue, a mosquito-borne disease caused by dengue virus (DENV), has threatened the world population due to its emergence and re-emergence in more than 100 countries [1]. DENV is an RNA virus of the Flaviviridae family, and presents 4 serotypes known as DENV-1, DENV-2, DENV-3, and DENV-4. The outcome of DENV infection can be an unnoticeable infection or be characterized by a spectrum of symptoms ranging from mild to severe dengue. The percentage of asymptomatic infection varies from 20 to 94% depending on the localization of the study and the virus serotype [2].

MicroRNAs (miRNA) are key elements in the regulation of gene expression, binding to mRNA transcripts and promoting their transitory sequestration or inducing their degradation [3]. They regulate a plethora of cellular processes, including immune response and host resistance to viral infection thus they are being used as biomarkers and therapeutic tools in viral diseases [4–8]. Some studies have evidenced that miRNAs play a role in DENV infection, through the targeting of innate immune response genes or binding to the DENV genome, meddling with dengue pathogenesis [9, 10].

Dengue is an endemic disease in Thailand with a high seroprevalence of anti-DENV IgG up to 84.5% of the population in central Thailand, 91% in northern Thailand, and 89% at national level with the highest incidence rates in people from 10 to 24 years old [11–13]. Therefore, dengue is firmly established in Thailand as a major public health concern, impacting nearly the entire population. Our hypothesis is that native Thai individuals without anti-DENV antibodies may represent a resistant population or possess a strong early immune response capable of rapidly suppressing DENV infection. This resistance may be driven, at least in part, by the antiviral and immune-regulating actions of particular miRNAs. We aimed to describe the miRNA expression profile of monocytes from people with or without antibodies against DENV, infected by DENV or mock, and assess their potential antiviral activity. Additionally, we investigated which biological routes and genes governed by these miRNAs may participate in the resistance, identifying new potential therapeutic targets to treat dengue pathogenesis.

## Materials and methods

### Volunteers’ enrollment, selection of DENV-resistant and DENV-susceptible individuals

We searched for individuals seronegative for antibodies against DENV, defined as resistant to DENV (RD). The study was approved by the Medical Ethics Committee of the Mahidol University, Thailand (COA N° MU-IRB 2012/180.1311). One hundred Thai adults (20–60 years) from Bangkok were enrolled and provided written informed consent. Ten ml of blood were sampled and their sera were tested by hemagglutinin inhibition assay. Hemagglutination inhibition test was performed following the protocol published by Clarke and Casals adapted by Duong et al. [14]. Due to the serological cross-reactivity between viruses from *Flaviviridae* family, sera were tested for NS1 DENV and Japanese Encephalitis Virus hemagglutination-inhibiting antibodies [14]. Seven DENV-seronegative and seven DENV-seropositive participants, susceptible to DENV (SD), were selected for further investigation.

### Infection assays in monocytes from DENV-resistant and DENV-susceptible individuals

To confirm the resistance or susceptibility to DENV infection, we monitored the efficiency of DENV replication in monocytes from the participants using a plaque assay. CD14 + PBMC were isolated by MACS^®^ technology (Miltenyi, https://www.miltenyibiotec.com) from 50 ml of blood re-sampled from the selected participants. The CD14 + monocytes were cultured in RPMI-1640 (Gibco, https://www.thermofisher.com) containing 10% human serum. Cell culture from each participant was split in two, one inoculated with DENV-2 isolate 16681 (MOI 1 pfu/cell) and the other for mock-infected control. Infections were set for 5 days and viral titer was measured from the supernatant every 24 h.

### miRNA microarray in monocytes from RD and SD participants

To evaluate miRNA expression profile, isolated monocytes from another 50 ml of blood of each RD and SD participant were mock and DENV-infected (DENV-2 isolate 16681, MOI 1 pfu/cell) for 6 h. Cells were pelleted, resuspended in RNA protect cell reagent (Qiagen, https://www.qiagen.com) and frozen at -80 °C. Total RNA was isolated from monocytes using miRNeasy kit (Qiagen) following the manufacturer’s instructions. RNA quantity was measured on Nanodrop spectrophotometer (ND-Nanodrop Technologies, https://www.mt.com) and RNA integrity number (RIN) was checked on Agilent Bioanalyzer using RNA 6000 Nano kit (Agilent, https://www.agilent.com). Sample RIN ranges were between 6.8 and 9.5. Low molecular weight RNAs were labeled with FlashTag Biotin HSR Labeling kit (Affymetrix, https://www.thermofisher.com) and hybridized overnight on GeneChip miRNA 4.0 arrays (Affymetrix). Microarrays were scanned using an Affymetrix GeneChip Scanner 3000. Quality control and data normalization were performed using Expression Console (Affymetrix). Afterwards, we performed normalization and signal to noise correction using Robust Multichip Average method (RMA) with R package Oligo [15], outlier detection and removal using principal component analysis (PCA), modeling and correcting for batch effects using surrogate variable analysis (SVA). We performed a differential expression analysis using the Limma data package from R [16] applying FDR (q-value) as a post-hoc statistical test.

### Virus strains for infections

We employed the following virus strains in our infection assays. The DENV-2 16681 isolate was used for infections of monocytes isolated from selected participants, conducted at Mahidol University. The parental DENV-2 16681 virus was originally isolated in 1964 from the serum of a dengue hemorrhagic fever/dengue shock syndrome patient in Bangkok, Thailand, and is widely recognized as a DENV-2 reference laboratory strain. The four serotypes of DENV were used for experiments with cells transfected with miRNA mimics, performed at Institut Pasteur, Paris. The DENV-1 KDH0026A strain and the DENV-3 KDH0010A strain were kindly provided by Dr. L. Lambrechts (Institut Pasteur, Paris). Both DENV-1 and DENV-3 are clinical isolates derived from human serum samples collected in 2010 from patients infected with DENV at the Kamphaeng Phet Provincial Hospital, Thailand [17]. The DENV-2 isolate B0301501 was generously provided by Dr. P. Dussart (Institut Pasteur, Cambodia) and was originally isolated from a 16-year-old male patient with dengue hemorrhagic fever in Phnom Penh Province, Cambodia. Finally, the DENV-4 strain VIMFH4 was derived from human serum collected in Burma (now Myanmar) in 1976 and was obtained from the Institut Pasteur Collection (P. Desprès, 2004).

### Cell lines for in vitro assays

HeLa cell line (Sigma-Aldrich 93021013, https://www.sigmaaldrich.com) are epithelial adherent cells derived from a cervical carcinoma from a 31-year-old black woman. HeLa cells are susceptible to DENV infection and therefore used as the base for miRNA-induced resistant model. Vero cell line (Sigma-Aldrich 84113001) are fibroblast-like cells, established from the kidney of a normal adult African Green monkey. Due to their high susceptibility to a wide range of virus we employed them to titrate DENV from infection supernatants. Both cell lines were cultured at 37 °C in a 5% CO_2_ atmosphere in Dulbecco’s modified Eagle’s medium (DMEM) supplemented with 10% (v/v) heat-inactivated Fetal Bovine Serum (FBS, Gibco, https://www.thermofisher.com) and Penicillin/Streptomycin (10,000 U/ml) antibiotic mix (Gibco).

### Transfection of mimics of selected miRNAs

HeLa cells were transfected in 5 replicates with miRNA mimics or miRVana mimic negative control (Ambion, https://www.thermofisher.com) at 50nM in OptiMEM (Gibco), and 0.8 µl Lipofectamine RNAiMAX (Invitrogen, https://www.thermofisher.com) as transfection reagent in OptiMEM. Transfections were done in a 24-well culture plate with 50,000 cells in a 500 µl total volume of DMEM supplemented with 10% (v/v) FBS.

### Infection of transfected cells

At 24 h post-transfection, HeLa cells were infected with DENV-1 KDH0026A strain at MOI 1 pfu/cell, DENV-2 isolate B0301501at MOI 4 pfu/cell, DENV-3 KDH0010A strain at MOI 5 pfu/cell, and DENV-4 isolate VIMFH4 at MOI 4 pfu/cell. Supernatants were harvested 48 h after infection and kept in -80 °C until virus titration. Transfected and infected cells were collected, washed in PBS, labeled with LIVE/DEAD stain (Invitrogen) then fixed in PFA 4% and permeabilized for 20 min in Cytofix/Cytoperm (BD Biosciences, https://www.bdbiosciences.com). Primary antibody 4G2, an in-house mouse anti-Flavivirus Envelope protein antibody, was incubated with cells for 30 min at 4 °C, then after 2 washes in Perm/Wash buffer (BD Biosciences) cells were incubated with a secondary Goat anti-mouse Alexa 488 (Invitrogen) antibody for 30 min at 4 °C. After washing in Perm/Wash buffer cells were resuspended in MACS buffer (Miltenyi) and analyzed on a MACSQuant cytometer (Miltenyi). Results were analyzed with Kaluza Software v2.1 (Beckman Coulter https://www.beckman.fr/) and statistical analysis was conducted with GraphPad Prism v9.5.0. Gating strategy is shown in Supplementary Fig. 1.

### Titration of transfected Hela cell supernatant by focus forming assay on Vero cells

Supernatants were diluted (serial 10-fold) in serum free DMEM, and 40 µl of viral dilution were incubated for 2 h at 37 °C/5% CO_2_ on 4 × 10^4^ Vero cells then covered with 115 µl of overlay media (DMEM + FBS 5% + Sodium Carboxymetylcellulose 1:1). Infection was stopped after 72 h by removing the overlay media and adding paraformaldehyde 4% to fix the cells. After PBS washes, cells were permeabilized and blocked in PBS containing 0.3% Triton and 5% FBS. Cells were then immuno-stained with a mouse 4G2 primary in-house antibody and goat anti-mouse IgG secondary antibody labelled with Alexa488 (Invitrogen A11029). The Focus Forming Units were then counted by an Immunospot^®^S6 Ultimate UV image analyzer. Results were exported from CTL software v6.0.0.2 and analyzed with GraphPad Prism v9.5.0. Virus titers were calculated and expressed as focus forming units (FFU) per ml.

### RNA-Seq of HeLa cells transfected with mir-155-5p mimic

HeLa cells were transfected by triplicates with 50 nM of miRVana miR-155-5p mimic or miRVana negative control and harvested 24 h post-transfection. Total RNA isolation was performed with Monarch^®^ Total RNA Miniprep Kit (NEB, https://www.neb.com) following the manufacturer’s instructions and extracts were kept in -80 °C until its use. RNA integrity number was evaluated and it was over 9.7 in all samples. RNA-Seq libraries were obtained from 500 ng of total RNA using TruSeq Stranded Total RNA library preparation kit (Illumina, https://www.illumina.com) and sequenced in a NextSeq 500/550 High Output Kit v2.5–75 cycles (Illumina) in single-end mode expecting 65 million reads per sample with a NextSeq 500 sequencer (Illumina). Raw reads were quality filtered and processed with Cutadapt v3.4 [18] and quality control was performed with FastQC v0.11.9 [19]. Filtered reads were aligned to the human transcriptome (Genome Reference Consortium Human Build 38) using RSEM pipeline with HISAT2 as aligner [20]. Once gene counts were calculated, a differential expression analysis was performed with DESeq2 [21]. Differentially expressed genes (DEG) in mimic-treated HeLa cells were cross-checked with miR-155-5p targets described in miRTarbase [22] and Tarbase [23] and with the Dengue-Human interaction database DenHunt [24] to find potential targets involved in the resistance to dengue. Additionally, an overrepresentation analysis was carried out in KOBAS-i [25] with DEGs described as miR-155-5p targets to highlight biological terms and signaling pathways from Reactome that might be involved in the resistance to DENV infection and replication.

## Results

### RD individuals show lower titers of DENV-2 production after in vitro infection

**Fig. 1:**
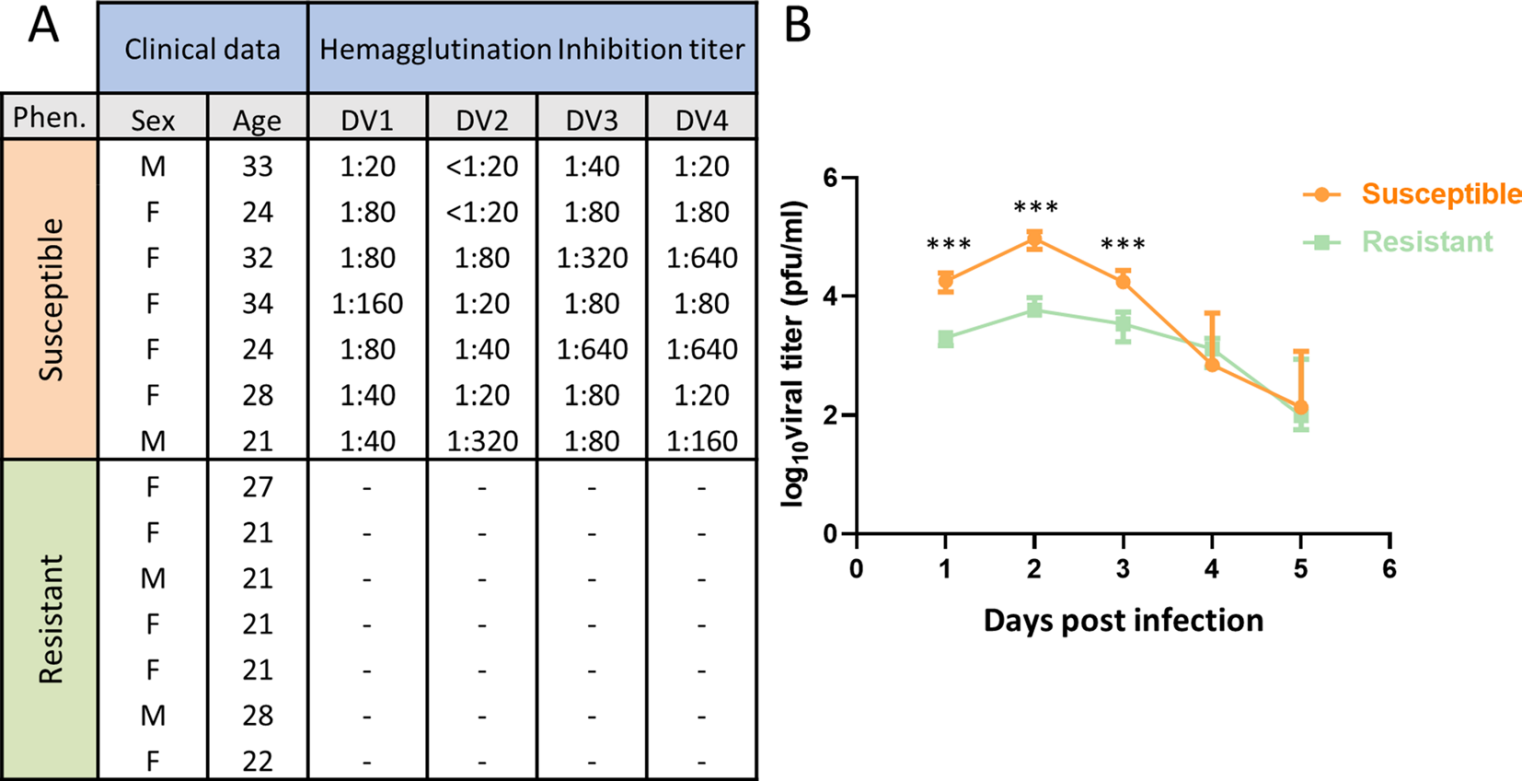
Phenotypic/immunological data of individuals, and viral titration of their monocytes after DENV-2 infection. (**A**) Gender, age and DENV antibodies detection by hemagglutination inhibition in the 2 groups. (**B**) Log10 viral titer median on days 1, 2, 3, 4, 5 post infection. Error bar figures inter-quartile range. Statistics (Mann-Whitney test) shows significant difference on days 1, 2 and 3 with p-value = 0.0006 (***) but no significant difference on days 4, 5

Seven individuals were chosen for the RD group regarding the absence of antibodies against all serotypes of DENV (Fig. 1A). The group of SD was constituted of seven anti-DENV seropositive individuals. The sex ratio was the same in both groups and average ages of 28 years in SD and 23 years in RD group (*p* = 0.057, range = 21–34 years), (Fig. 1A).

To investigate differential susceptibility to infection between the 2 groups, donor monocytes were inoculated with DENV-2 at MOI 1 pfu/cell. The efficiency of replication was monitored within 5 days post-infection, showing a mean viral titer in the SD group 9-fold higher than the RD group at 1-day post-infection (dpi), 13-fold higher at 2 dpi, and 5-fold higher at 3 dpi (Fig. 1B). Therefore, monocytes from RD participants yield a reduced viral progeny.

### Some miRNAs show differential expression in the context of DENV resistance

To explore the miRNA role in the resistance to DENV we performed a miRNA chip hybridization assay for mock-infected cells and for DENV-infected cells in both RD and SD groups. After data quality control two samples were removed, one from RD mock-infected cells due to poor hybridization and one outlier from SD infected group. We performed a pairwise comparison of miRNA expression between the experimental groups and miRNA were considered differentially expressed (DE) with absolute values of fold change (FC) > 1.25 and q-value < 0.05. The list of DE miRNAs in each comparison are given in supplementary Tables ST1, ST2, ST3, and ST4.

**Fig. 2:**
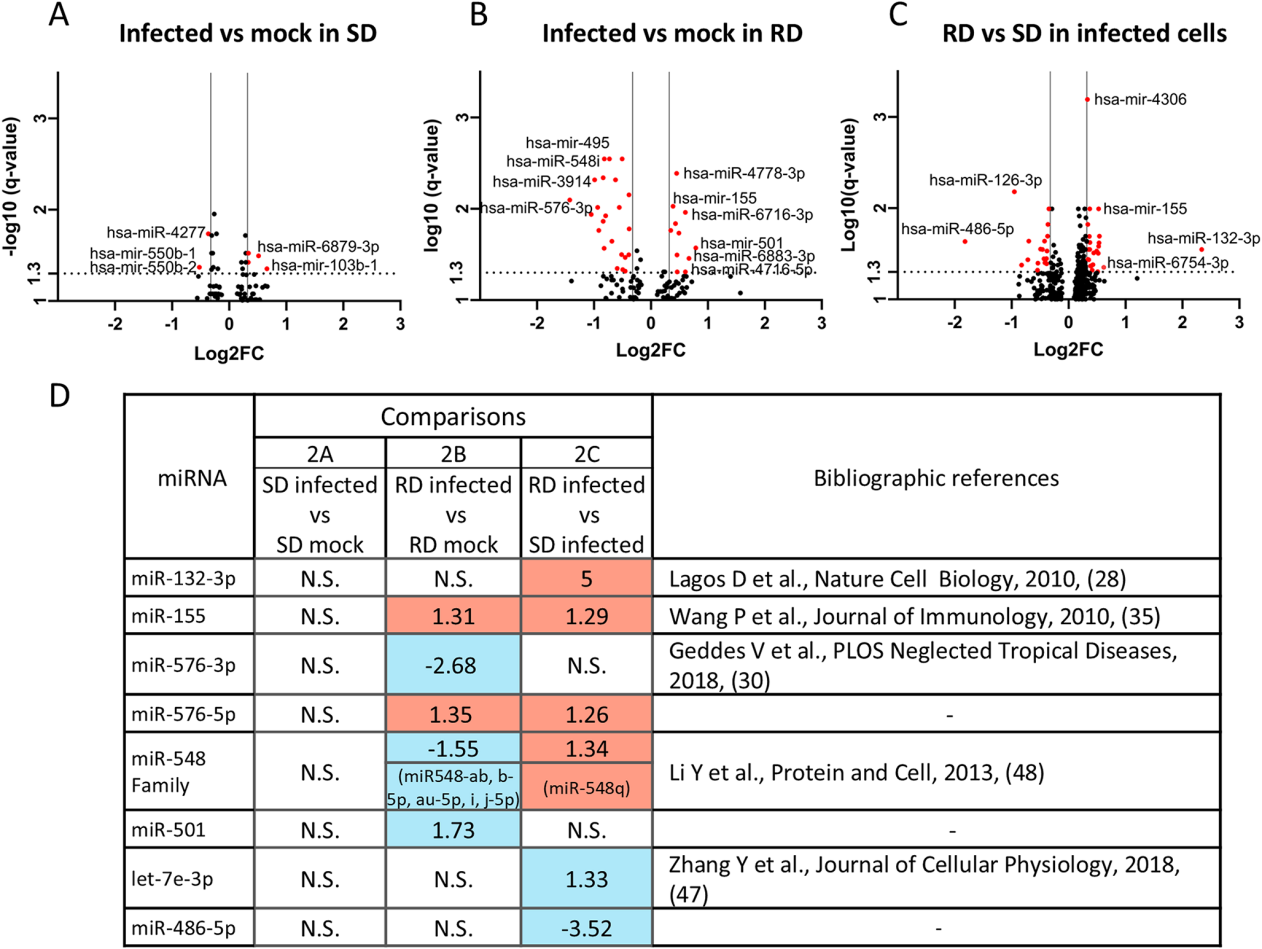
Representation of differentially expressed miRNA. (**A to C**): Volcano plots showing changes in miRNA expression levels according groups (RD = Resistant to dengue viral infection, SD = Susceptible to dengue viral infection). Red dots denote significantly differentially expressed miRNA (q-value < 0.05, Log10 q-value > 1.3) whose expression changed by more than 1.25-fold. Vertical line represents log2FC ± 0.32 threshold, and horizontal dotted line represents statistical significance threshold (q-value = 0.05). (**D**) Overall view of expression of selected miRNA. Salmon boxes represent overexpression while blue boxes mean reduction of expression. In the center of each colored box is the Fold Change (+/-) N.S. Not significant: q-value ;> 0.05

First, comparing DENV-infected vs. mock-infected monocytes in the SD group we found seven miRNAs, including miR-103b-1, miR-6879-3p, and miR-550b-1, that may participate in the cell response to infection process (Fig. 2A, Supplementary Table ST1), ruling them out as exclusive mechanisms of resistance in the RD group. Secondly, we evaluated the alteration of miRNAs expression upon infection in monocytes from RD group. We found 24 downregulated and 10 upregulated miRNAs in DENV-infected cells, including miR-576-3p (FC=-2.6, q-value = 8.0 × 10^− 3^) and a few members of miR-548 family (548n, 548b-5p, 548j-5p, 548i) among the most repressed ones. Conversely, miR-501 (FC = 1.73, q-value = 2.7 × 10^− 2^), miR-576-5p (FC = 1.35, q-value = 1.5 × 10^− 2^), and miR-155 (FC = 1.31, q-value = 9.37 × 10^− 3^) were upregulated in DENV-infected monocytes of RD participants (Fig. 2B, Supplementary Table ST2). This miRNA expression pattern observed in RD could play a role in the resistance mechanisms.

Subsequently, we studied the differences between infected monocytes from RD group vs. SD group, as this comparison shows DE miRNAs that may participate in viral blocking pathways present in RD individuals (Fig. 2C, Supplementary Table ST3). We found 19 downregulated and 23 upregulated miRNAs in RD, with a remarkable 5-fold increase in the expression of miR-132-3p (FC = 5.0, q-value = 2.8 × 10^− 2^). Among upregulated miRNAs, it is worth noting two miRNAs described in the literature for their role in immune response, let-7e-3p (FC = 1.33 q-value = 4.7 × 10^− 2^) and miR-155 (FC = 1.29, q-value = 1.0 × 10^− 2^). In addition, miR-576-5p (FC = 1.27, q-value = 3.6 × 10^− 2^) and the aforementioned miR-155 are upregulated as in the DENV-infected vs. mock-infected cells comparison in the RD group (Fig. 2B), suggesting a protective role in DENV infection. Additionally, we obtained 17 DE miRNAs comparing mock-infected monocytes from RD to SD groups (Supplementary Table ST4), to find some miRNAs that could play a role in the resistance to the infection in the monocytes basal state. We found that miR-576-3p (FC = 2.23, q-value = 1.7 × 10^− 4^) along with three members of miR-548 family (548i, 548au-5p, and 548b-5p) are overexpressed in RD.

Finally, we summarized the most interesting DE miRNA based on the FC in three comparisons, including published studies that support a potential role in the pathogenesis of infectious diseases and antiviral immune response (Fig. 2D). Therefore, we chose miR-132-3p, miR-576-3p, and miR-155-5p as candidates for performing functional assays and assessing their role as antiviral miRNAs. In the case of miR-155, we selected the − 5p arm since it is preferred in the biogenesis process and regulate up to 220 gene targets defined in miRTarbase (4 gene targets for − 3p arm).

### Mir-155-5p upregulation decreases DENV infection in HeLa cells

**Fig. 3:**
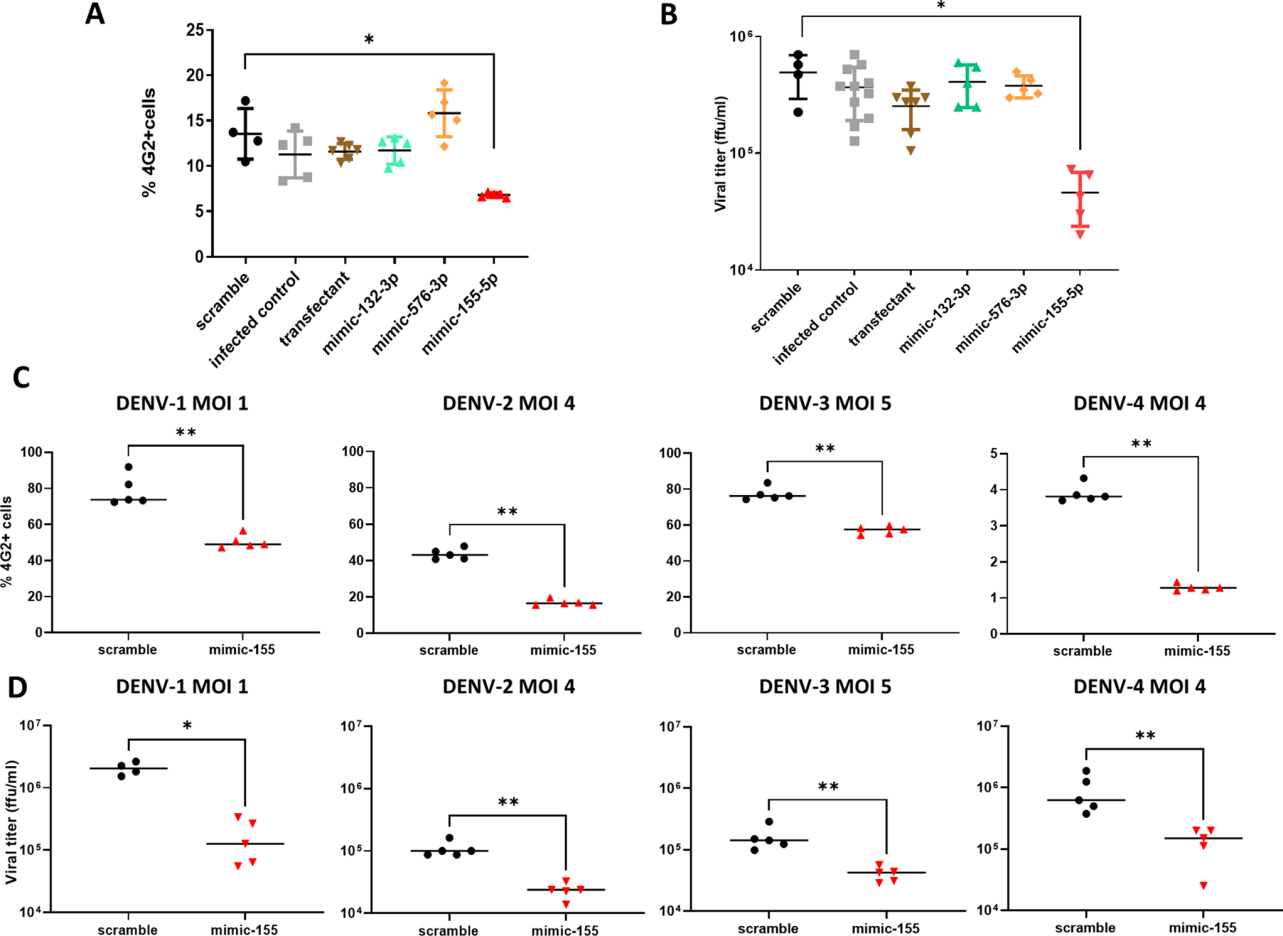
Functional study to show effect of miRNA mimic transfection on DENV infection. (**A**) Cytometry on DENV-1 infected HeLa cells, 48 h post infection, transfection 24 h, and (**B**) viral titration of supernatants of those cells. (**C**) Cytometry on DENV-1, DENV-2, DENV-3, DENV-4 infected HeLa cells, 48 h post infection, transfection 24 h. (**D**) Viral titration of supernatants of those cells. Statistics: Mann-Whitney non-parametric tests ** *p* = 0,0079; * *p* = 0,0159

We performed functional assays with the selected miRNA candidates to investigate their effect in the infection and viral production. For this purpose, we performed a transient upregulation of miR-132-3p, miR-576-3p, and miR-155-5p through the transfection of mimics of each miRNA. The transient upregulation of miR-155-5p caused a significant reduction in infection rate, although miR-576-3p and miR-132-3p overexpression did not affect intracellular detection of DENV E protein (Fig. 3A). These results are congruent with the more than one log10 reduction in viral titer upon miR-155-5p mimic treatment (Fig. 3B).

To verify if miR-155-5p upregulation produced an antiviral effect with all DENV serotypes, we infected with DENV-1, DENV-2, DENV-3, and DENV-4 mimic-transfected HeLa cells, either with miR-155-5p mimic or a scramble miRNA (mimic negative control). We observed a reduction in the rate of infection 48 h post-infection (hpi) of 36% in DENV-1, 62% in DENV-2, 26% in DENV-3, and 68% in DENV-4 upon overexpression of miR-155-5p (Fig. 3C). The reduction of viral titers supports the data of infection rates, decreasing a 92% for DENV-1, 79% for DENV-2, 75% for DENV-3 and 85% for DENV-4 48hpi, suggesting that the overexpression of miR-155-5p elicits an antiviral response in HeLa cells.

### Mir-155-5p shapes transcriptomic landscape restricting dengue pathogenesis

**Fig. 4:**
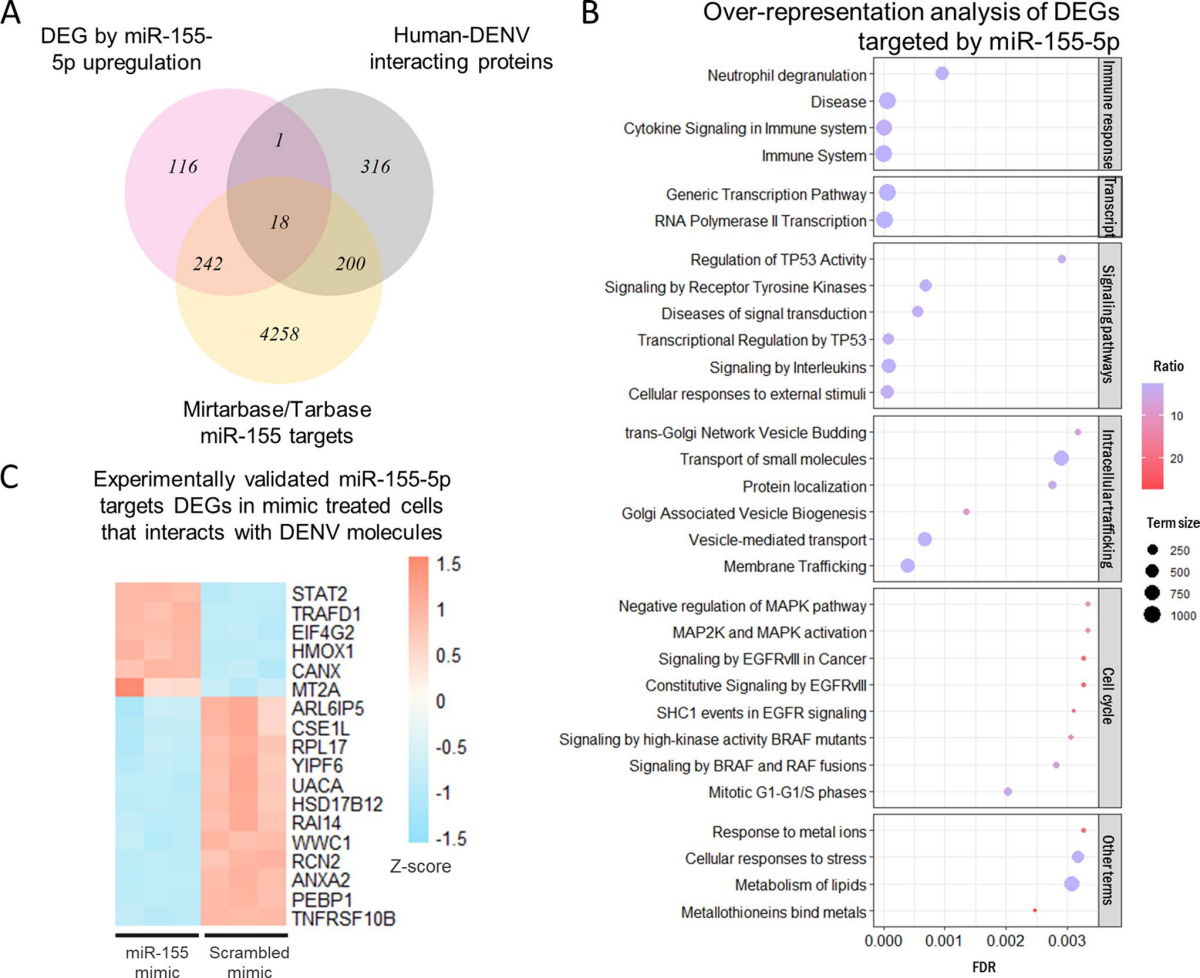
RNA-Seq in HeLa cells transfected with miR-155-5p mimic or scrambled mimic. (**A**) Venn diagram representing DEG in Hela cell transfection (pink), DENV-Human protein interactions registered in DENVHunt Database (grey) and experimentally validated targets of miR-155-5p from miRTarbase and Tarbase (yellow). (**B**) Bubble plot representing biological terms from Reactome enriched with the 260 DEGs targeted by miR-155-5p as registered in miRTarbase and Tarbase. The 30 terms with lower FDR are shown. Bubble size refers to the number of genes that compose the biological term. Chromatic scale indicates the percentage of genes that are DEG in each biological term. Transcript. = Transcription. (**C**) Heatmap of the 18 DEG in Hela cell transfection experiment defined as experimentally validated targets of miR-155-5p and that interact with DENV proteins. Chromatic scale represents Z-score of normalized counts

With a view to describe what processes and genes could be altered by miR-155-5p upregulation, we performed an RNA-Seq on Hela cells 24 h after mimic transfection. We identified in miR-155-5p mimic transfected cells 265 downregulated DEGs (FC<-2, FDR < 0.05) and 112 overexpressed DEGs (FC > 2, FDR < 0.05) (Supplementary Table 5), of which 260 DEGs were defined as miR-155-5p targets in miRNA-mRNA interaction databases (Fig. 4A). Of those, 209 genes are downregulated and presumably under the direct repression brought on by mir-155-5p overexpression. The over-representation analysis showed that a total of 115 (FDR < 0.05) biological terms from the Reactome database are enriched with the 260 DEGs targeted by miR-155-5p (Fig. 4B, Supplementary Table 6), and thus influenced by miR-155-5p upregulation. These genes controlled by miR-155-5p are involved in immune response processes, transcriptional activities, the regulation of TP53 signaling, in vesicle and molecule trafficking and cell cycle regulating-signaling (MAPK, EGFR, BRAF, AKT) pathways. In addition, those DEG participates in processes regulated by interferon signaling, endocytosis, apoptosis, or mTOR pathway (Supplementary Table 6). It is compelling that 18 DEGs targeted by miR-155-5p are described as interactors of DENV proteins (Fig. 4C). Downregulated genes (ANXA2, PEPB1, TNFRSF10B, RPL17, HSD17B12) are explained because of direct repression of miR-155-5p, although others such as HMOX1, MT2A, TRAFD1 or EIF4G2 could be overexpressed by an indirect mechanism of upregulation by this miRNA.

## Discussion

We have demonstrated that the expression of some miRNAs is altered in individuals with resistance to DENV infection. This resistance was defined as the absence of anti-DENV specific antibodies in their sera, based on demographic and epidemiological data for native Thai population, and later supported by a lower DENV titer in monocytes of RD participants in the infection assay. The comparison between RD and SD groups, their monocytes being DENV-2 or mock-infected, showed several differentially expressed miRNAs. The most overexpressed miRNA is miR-132-3p, being 5-fold higher in the infected monocytes from the RD group vs. SD. miR-132 is known to be over-expressed in monocytes early after infection, as miR-155 [26, 27] that act in the modulation of innate immune response, through the repression of the p300 transcriptional co-activator [28]. In the context of the RD group, miR-576-3p is downregulated upon infection whereas it is upregulated when compared to the SD monocytes in the absence of infection. This miRNA is reported to be a regulator of antiviral response, sensitizing cells to viral replication of the Vesicular Stomatitis Virus [29] and a proviral factor in Oropouche virus infection [30]. Our differential expression study shows that miR-576-3p is 2.6-fold less expressed in DENV-infected cells vs. mock-infected from the RD individuals, which could be a mechanism of protection upon infection. However, our results do not display any antiviral activity of these miRNA in the functional assays. If these miRNAs can play a role in the resistance to DENV infection/pathogenesis in other cell types or at a systemic level, this is out of the reach of our study but cannot be ruled out.

Lastly, miR-155 shows a pattern of overexpression in the RD individuals upon infection (FC = 1.31) and in infected monocytes of RD versus SD participants (FC = 1.29). In our functional assays, miR-155-5p upregulation is the only miRNA that shows an antiviral effect on DENV infection. This miRNA, specifically its − 5p arm, has been extensively reported to regulate immune processes which is in agreement with the multiple immune related processes that we obtained in the RNA-Seq [26, 31–34]. For instance, miR-155-5p strengthens antiviral immunity and elicits a more effective innate and adaptive immune response [26]. It directly represses protein levels of suppressor of cytokine signaling 1 (SOCS1), which is a negative regulator of the TLR pathway and type I IFN signaling [35] enhancing innate response. Accordingly, Su et al. reported that the upregulation of miR-155-5p inhibits a key transcriptional repressor of HMOX1, which promotes the activation of antiviral interferon responses [36]. Our study results reproduce the data from this work. This miRNA also plays an important role in B cell immune function by targeting AID to regulate antibody class-switching and somatic hypermutation [37] while it helps CD4 T cells to differentiate towards a Th1 response [38].

Other biological processes are enriched in the genes targeted by miR-155-5p as regulation of TP53, interferon signaling, or apoptosis. The presence of the DENV capsid protein induces ribosomal stress and apoptosis in human neurons through TP53 sensing [39]. Also, it has been reported that DENV infection promotes the activation of TP53-mediated apoptosis, assisting in the reduction of immune response and inflammation and thus favoring viral propagation [40]. The control that miR-155-5p may exert on these pathways, for instance repressing the expression of TNFRSF10B, a TNF-receptor that mediates apoptosis, or PEBP1 that enhances the induction of p53-modulated apoptosis [41], could lead to a stronger innate response, counteracting the DENV infection effect. In addition, their interactors are DENV2-NS2A and DENV2-prM proteins respectively, suggesting that DENV-host required interactions could be hampered.

Moreover, terms such as vesicle-mediated transport, biogenesis, budding, and endocytosis are enriched in miR-155-5p gene targets. The relevance of vesicle trafficking has been established in DENV infection, not only in DENV entry [42] to the cell but also in viral protein transport to membranes and the accumulation of lipid droplets for virion assembly [43]. Regarding that, the recruitment of fatty acids synthases to sites of viral replication is necessary to complete the assembly and maturation. Interestingly, HSD17B12, which is a core interactor of the very-long-chain fatty acid synthesis pathway, interacts with DENV-NS3 to be recruited towards viral factories and its knockdown impairs DENV and other flavivirus production [44]. Furthermore, the genes products of ANXA2 and RCN2, which directly interacts with NS1 and NS3 DENV proteins respectively, also participates in this process. ANXA2 is involved in filopodia formation, required for a successful DENV infection, and its knockdown reduces DENV-2 viral production and infection [45]. RCN2 is a chaperone localized in the endoplasmic reticulum that interacts with viperin, an interferon stimulated gene that impedes viral particle assembly [46]. We show that the upregulation of miR-155-5p causes the repression of HSD17B12, ANXA2, and RCN2 in HeLa cells and that may explain the reduction in viral titers. However, our study presents some limitations as the reduced participant number, lack of previous data confirming DENV infection or the in vitro transfection model. This experiment should be reproduced in vivo to confirm the miR-155 effect on DENV restriction. We want to mention that miRNA activity may be one of the factors that limit or controls DENV infection, likely working in conjunction with complementary processes such as monocyte restriction or a robust early immune response. These mechanisms warrant further investigation in future research projects. [35, 47, 48].

Taken together we show that the monocytes from individuals resistant to dengue infection present constraints to DENV infection and replication. They present a miRNA transcriptional signature related to miR-155, miR-132-3p, miR-576-3p, miR-576-5p and a few miR-548 family members. The upregulation of miR-155-5p in vitro partially inhibits viral infection and production through a number of biological processes as enhancing interferon response, regulating TP53 activity, apoptosis, and vesicle trafficking. We propose that the research on miR-155-5p targets (HSD17B12, ANXA2, RCN2, TNFRSF10B, PEBP1) could be of use in the development of new therapeutic agents to treat dengue infection.

## Electronic supplementary material

Below is the link to the electronic supplementary material.

Supplementary Material 1

Supplementary Material 2

Supplementary Material 3

Supplementary Material 4

Supplementary Material 5

Supplementary Material 6

## Data Availability

Sequence data that support the findings of this study have been deposited in Array Express with accession number E-MTAB-13628 (https://www.ebi.ac.uk/biostudies/studies/E-MTAB-13628).

## Abbreviations

DENV: Dengue virus
RD: Resistant to Dengue
SD: Susceptible to Dengue
HSD17B12: 17β-hydroxysteroid dehydrogenase type 12
ANXA2: Annexin 2
RCN2: Reticulocalbin 2
TNFRSF10B: Tumor necrosis factor receptor superfamily member 10b
PEBP1: Phosphatidylethanolamine binding protein 1
MOI: Multiplicity of infection
RIN: RNA integrity number
FDR: False discovery rate
DMEM: Dulbecco’s modified Eagle’s medium
PBS: Phosphate buffered saline
FBS: Fetal bovine serum
FFU: Focus forming unit
DEG: Differentially expressed gene
FC: Fold change
ST: Supplementary table
MAPK: Mitogen activated protein kinase
EGFR: Epidermal growth factor receptor
BRAF: B-Raf proto-oncogene, serine/threonine kinase
AKT: Ak strain transforming
RPL17: Ribosomal protein L17
HMOX1: Heme oxygenase 1
MT2A: Metallothionein 2 A
TRAFD1: TRAF-type zinc finger domain containing
EIF4G2: Eukaryotic translation initiation factor 4 gamma 2
SOCS1: Suppressor of cytokine signaling 1
TLR: Toll-like receptor
AID: Activation-induced cytidine deaminase
TP53: Tumor protein 53
