# Supplementary material for: miRNome analysis reveals mir-155-5p as a protective factor to dengue infection in a resistant Thai cohort
Source: Med Microbiol Immunol. 2025 Feb 20;214(1):13. doi: 10.1007/s00430-025-00821-7 (PMC11842423; doi:10.1007/s00430-025-00821-7)
Supplement: Supplementary file 3 — Supplementary Material 3 [file 430_2025_821_MOESM3_ESM.docx]

ST1. Supplementary table 1. Up and down-regulated miRNAs of **infected versus mock cells in SD** group (q-value<0.05). In dark grey, |FC|>1,5 and in light grey 1,5>|FC|>1,25

| miRNA | Fold Change | Log_2_FC | p-value | q-value |
| --- | --- | --- | --- | --- |
| hsa-mir-103b-1 | 1,58 | 0,66 | 2,78E-04 | 4,44E-02 |
| hsa-miR-6879-3p | 1,43 | 0,52 | 1,24E-04 | 3,20E-02 |
| hsa-mir-4497 | 1,26 | 0,34 | 9,16E-05 | 2,99E-02 |
| hsa-mir-338 | 1,26 | 0,33 | 1,65E-04 | 3,76E-02 |
| hsa-miR-4277 | -1,29 | -0,37 | 3,17E-05 | 1,83E-02 |
| hsa-mir-550b-1 | -1,44 | -0,52 | 2,40E-04 | 4,27E-02 |
| hsa-mir-550b-2 | -1,44 | -0,52 | 2,40E-04 | 4,27E-02 |

ST2. Supplementary table 2. Up and down-regulated miRNA of **infected versus mock cells in RD** group (q-value<0.05) . In dark grey, |FC|>1,5 and in light grey 1,5>|FC|>1,25

| miRNA | Fold Change | Log_2_FC | p-value | q-value |
| --- | --- | --- | --- | --- |
| hsa-mir-501 | 1,73 | 0,79 | 1,84E-04 | 2,68E-02 |
| hsa-miR-6883-3p | 1,59 | 0,67 | 3,28E-04 | 3,49E-02 |
| hsa-miR-6716-3p | 1,52 | 0,61 | 3,64E-05 | 1,09E-02 |
| hsa-miR-4716-5p | 1,52 | 0,6 | 5,78E-04 | 4,90E-02 |
| hsa-miR-6870-3p | 1,41 | 0,49 | 1,08E-04 | 1,84E-02 |
| hsa-miR-5699-3p | 1,38 | 0,46 | 5,81E-04 | 4,90E-02 |
| hsa-miR-4778-3p | 1,37 | 0,45 | 3,21E-06 | 4,09E-03 |
| hsa-miR-576-5p | 1,35 | 0,43 | 6,82E-05 | 1,45E-02 |
| hsa-mir-155 | 1,31 | 0,39 | 2,02E-05 | 9,37E-03 |
| hsa-miR-664a-3p | 1,27 | 0,35 | 9,62E-05 | 1,73E-02 |
| hsa-mir-208a | -1,3 | -0,38 | 8,14E-05 | 1,66E-02 |
| hsa-mir-1252 | -1,31 | -0,39 | 1,10E-05 | 7,02E-03 |
| hsa-mir-3686 | -1,31 | -0,39 | 2,62E-04 | 3,19E-02 |
| hsa-miR-671-5p | -1,37 | -0,45 | 5,38E-04 | 4,81E-02 |
| hsa-miR-6832-5p | -1,37 | -0,45 | 3,14E-04 | 3,41E-02 |
| hsa-miR-3134 | -1,41 | -0,49 | 5,00E-04 | 4,63E-02 |
| hsa-mir-4275 | -1,42 | -0,5 | 1,30E-06 | 2,84E-03 |
| hsa-mir-302d | -1,43 | -0,51 | 2,51E-04 | 3,17E-02 |
| hsa-mir-548n | -1,47 | -0,55 | 3,03E-05 | 9,64E-03 |
| hsa-mir-8084 | -1,51 | -0,59 | 4,62E-04 | 4,50E-02 |
| hsa-miR-548b-5p | -1,54 | -0,62 | 6,60E-06 | 4,81E-03 |
| hsa-miR-651-3p | -1,61 | -0,68 | 1,51E-04 | 2,27E-02 |
| hsa-mir-495 | -1,65 | -0,73 | 1,67E-06 | 2,84E-03 |
| hsa-miR-548j-5p | -1,74 | -0,8 | 4,44E-05 | 1,19E-02 |
| hsa-miR-548i | -1,76 | -0,82 | 7,89E-07 | 2,84E-03 |
| hsa-mir-517a | -1,77 | -0,82 | 1,91E-04 | 2,70E-02 |
| hsa-mir-363 | -1,79 | -0,84 | 4,48E-06 | 4,56E-03 |
| hsa-mir-4679-1 | -1,79 | -0,84 | 6,09E-05 | 1,37E-02 |
| hsa-mir-4679-2 | -1,79 | -0,84 | 6,09E-05 | 1,37E-02 |
| hsa-mir-620 | -1,89 | -0,92 | 9,19E-05 | 1,73E-02 |
| hsa-mir-520f | -1,91 | -0,93 | 2,96E-05 | 9,64E-03 |
| hsa-miR-3914 | -1,98 | -0,99 | 6,33E-06 | 4,81E-03 |
| hsa-miR-129-1-3p | -2,07 | -1,05 | 4,06E-05 | 1,15E-02 |
| hsa-miR-576-3p | -2,68 | -1,42 | 1,57E-05 | 8,02E-03 |

ST3. Supplementary table 3. Up and down-regulated miRNA of **RD versus SD group in infected** cells (q-value<0.05) . In dark grey, |FC|>1,5 and in light grey 1,5>|FC|>1,25

| miRNA | Fold Change | Log_2_FC | p-value | q-value |
| --- | --- | --- | --- | --- |
| hsa-miR-132-3p | 5,05 | 2,34 | 4,40E-04 | 2,84E-02 |
| hsa-miR-6754-3p | 1,53 | 0,62 | 1,41E-03 | 4,46E-02 |
| hsa-miR-5699-3p | 1,45 | 0,54 | 1,21E-04 | 2,03E-02 |
| hsa-miR-187-3p | 1,45 | 0,53 | 2,01E-04 | 2,40E-02 |
| hsa-miR-4720-3p | 1,44 | 0,53 | 3,64E-05 | 1,02E-02 |
| hsa-miR-5010-3p | 1,44 | 0,53 | 2,64E-04 | 2,62E-02 |
| hsa-miR-139-5p | 1,43 | 0,52 | 5,83E-04 | 3,13E-02 |
| hsa-miR-3184-3p | 1,43 | 0,51 | 1,87E-03 | 4,98E-02 |
| hsa-miR-6882-3p | 1,41 | 0,5 | 4,70E-04 | 2,95E-02 |
| hsa-miR-4697-3p | 1,36 | 0,44 | 5,35E-04 | 3,10E-02 |
| hsa-miR-4714-5p | 1,35 | 0,43 | 1,18E-03 | 4,16E-02 |
| hsa-let-7e-3p | 1,33 | 0,42 | 1,67E-03 | 4,79E-02 |
| hsa-miR-4288 | 1,33 | 0,41 | 1,26E-03 | 4,24E-02 |
| hsa-mir-3689f | 1,31 | 0,39 | 1,32E-03 | 4,30E-02 |
| hsa-mir-4659a | 1,3 | 0,38 | 1,93E-04 | 2,37E-02 |
| hsa-miR-3179 | 1,29 | 0,37 | 3,81E-04 | 2,84E-02 |
| hsa-mir-155 | 1,29 | 0,37 | 3,43E-05 | 1,02E-02 |
| hsa-miR-509-3p | 1,29 | 0,37 | 1,27E-04 | 2,04E-02 |
| hsa-miR-1293 | 1,28 | 0,35 | 8,04E-04 | 3,68E-02 |
| hsa-miR-134-5p | 1,28 | 0,35 | 4,20E-04 | 2,84E-02 |
| hsa-miR-576-5p | 1,27 | 0,34 | 7,59E-04 | 3,65E-02 |
| hsa-miR-153-5p | 1,26 | 0,34 | 7,21E-05 | 1,51E-02 |
| hsa-mir-4306 | 1,26 | 0,33 | 3,07E-07 | 6,41E-04 |
| hsa-mir-5585 | -1,27 | -0,34 | 3,00E-05 | 1,02E-02 |
| hsa-mir-338 | -1,28 | -0,36 | 6,89E-05 | 1,51E-02 |
| hsa-mir-6858 | -1,29 | -0,37 | 1,18E-04 | 2,03E-02 |
| hsa-miR-6726-3p | -1,3 | -0,38 | 4,90E-04 | 2,95E-02 |
| hsa-mir-378e | -1,31 | -0,38 | 1,13E-03 | 4,07E-02 |
| hsa-mir-188 | -1,31 | -0,39 | 7,28E-04 | 3,58E-02 |
| hsa-mir-2392 | -1,32 | -0,4 | 1,01E-03 | 3,99E-02 |
| hsa-miR-4768-3p | -1,33 | -0,42 | 9,97E-04 | 3,97E-02 |
| hsa-miR-548q | -1,34 | -0,42 | 1,65E-04 | 2,29E-02 |
| hsa-miR-410-3p | -1,35 | -0,43 | 7,38E-04 | 3,59E-02 |
| hsa-miR-6879-3p | -1,37 | -0,46 | 4,20E-04 | 2,84E-02 |
| hsa-mir-7154 | -1,4 | -0,49 | 3,28E-04 | 2,81E-02 |
| hsa-miR-1324 | -1,45 | -0,54 | 1,07E-03 | 4,01E-02 |
| hsa-mir-103b-1 | -1,46 | -0,54 | 1,68E-03 | 4,79E-02 |
| hsa-miR-4671-5p | -1,62 | -0,7 | 1,56E-04 | 2,29E-02 |
| hsa-miR-5094 | -1,64 | -0,72 | 7,83E-04 | 3,68E-02 |
| hsa-miR-216b-5p | -1,77 | -0,82 | 1,22E-03 | 4,21E-02 |
| hsa-miR-126-3p | -1,93 | -0,95 | 1,10E-05 | 6,59E-03 |
| hsa-miR-486-5p | -3,53 | -1,82 | 1,80E-04 | 2,33E-02 |

ST4. Supplementary table 4. Up and down-regulated miRNA of **RD versus** **SD group in mock** cells (q-value<0.05) . In dark grey, |FC|>1,5 and in light grey 1,5>|FC|>1,25

| miRNA | Fold change | Log_2_FC | p-value | q-value |
| --- | --- | --- | --- | --- |
| hsa-miR-576-3p | 2,23 | 1,16 | 1,72E-04 | 4,19E-02 |
| hsa-mir-520f | 1,89 | 0,92 | 3,68E-05 | 2,25E-02 |
| hsa-mir-620 | 1,85 | 0,89 | 1,33E-04 | 4,07E-02 |
| hsa-mir-363 | 1,58 | 0,66 | 7,68E-05 | 2,89E-02 |
| hsa-miR-548i | 1,58 | 0,66 | 1,47E-05 | 1,73E-02 |
| hsa-miR-548au-5p | 1,56 | 0,65 | 1,50E-04 | 4,08E-02 |
| hsa-miR-548b-5p | 1,49 | 0,57 | 1,77E-05 | 1,73E-02 |
| hsa-mir-495 | 1,48 | 0,56 | 4,34E-05 | 2,35E-02 |
| hsa-mir-302d | 1,44 | 0,53 | 1,93E-04 | 4,34E-02 |
| hsa-mir-4275 | 1,36 | 0,45 | 6,84E-06 | 1,67E-02 |
| hsa-miR-4999-3p | -1,5 | -0,58 | 1,95E-04 | 4,34E-02 |
| hsa-miR-4735-5p | -1,56 | -0,64 | 2,24E-04 | 4,76E-02 |
| hsa-miR-6716-3p | -1,58 | -0,66 | 1,27E-05 | 1,73E-02 |
| hsa-miR-6891-3p | -1,79 | -0,84 | 9,29E-05 | 3,02E-02 |
| hsa-let-7f-1-3p | -1,96 | -0,97 | 5,23E-05 | 2,55E-02 |
| hsa-miR-4789-3p | -1,96 | -0,97 | 5,76E-06 | 1,67E-02 |
| hsa-miR-6884-3p | -2,09 | -1,06 | 6,11E-05 | 2,62E-02 |
