# Supplementary figures and images for: miRNome analysis reveals mir-155-5p as a protective factor to dengue infection in a resistant Thai cohort
Source: Med Microbiol Immunol. 2025 Feb 20;214(1):13. doi: 10.1007/s00430-025-00821-7 (PMC11842423; doi:10.1007/s00430-025-00821-7)

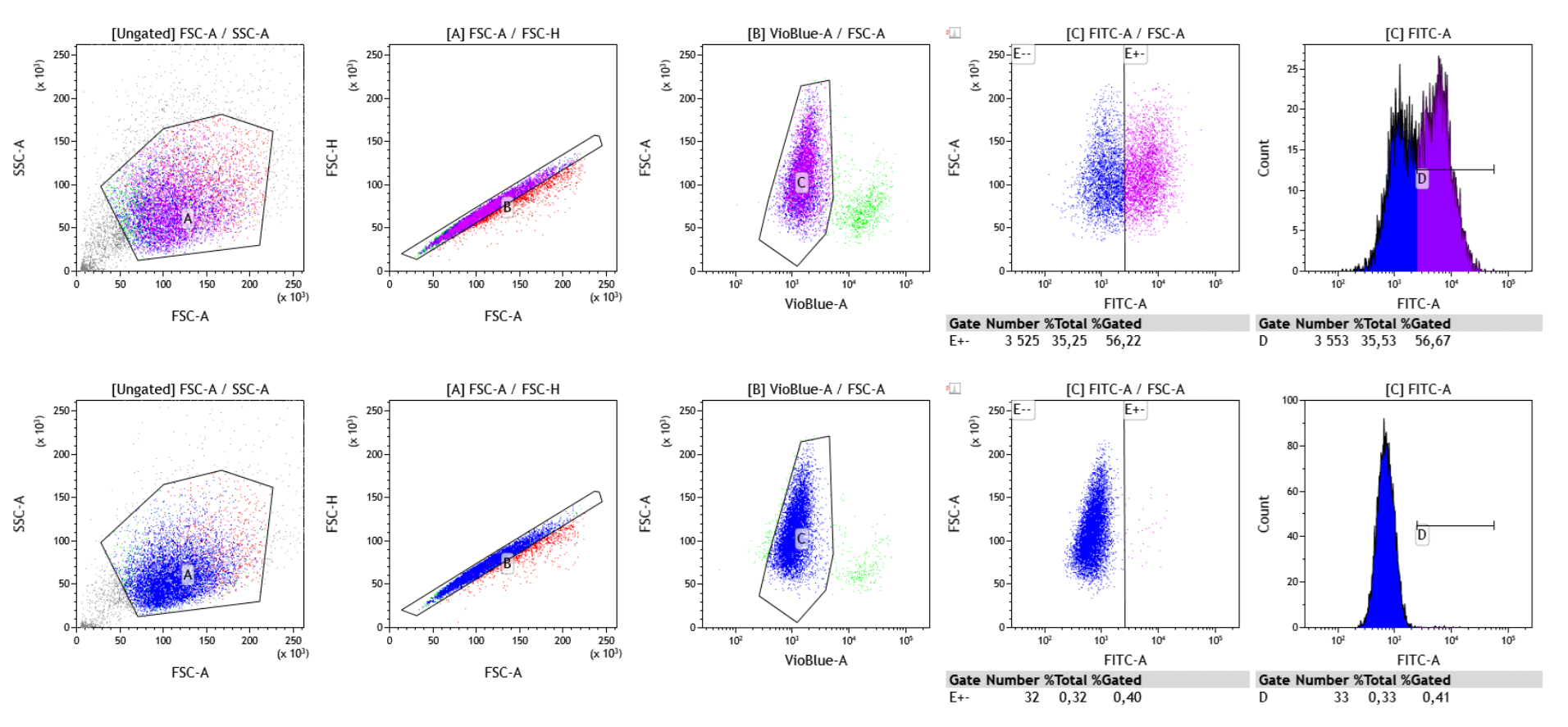

Supplement: Supplementary file 5 — Supplementary Material 5 [file 430_2025_821_MOESM5_ESM.tif]
