# Supplementary material for: miRNome analysis reveals mir-155-5p as a protective factor to dengue infection in a resistant Thai cohort
Source: Med Microbiol Immunol. 2025 Feb 20;214(1):13. doi: 10.1007/s00430-025-00821-7 (PMC11842423; doi:10.1007/s00430-025-00821-7)
Supplement: Supplementary file 6 — Supplementary Material 6 [file 430_2025_821_MOESM6_ESM.docx]

*Supplementary Figure 1. Gating strategy used to calculate percentage of infection in HeLa cells transfected with miRNA mimics. This is a representative experiment of HeLa cells transfected with miR-155-5p mimic following infection with DENV-1 at MOI 1. Panels above represent infected HeLa cells while panels below represent an uninfected sample. Gates starting from parental plot (left) show (1) Total events (SSC-A vs FSC-A) -> (2) Singlets (FSC-H vs FSC-A) -> (3) Alive cells (FSC-A vs Vioblue-A) -> (4) Alexa-488 positive cells (FSC-A vs FITC-A). Final plot show histogram for FITC-A channel, showing the intensity and threshold established for positivity of 4G2+ goat anti mouse Alexa-488 antibody.*
